# TGFβ2 is a prognostic‐related biomarker and correlated with immune infiltrates in gastric cancer

**DOI:** 10.1111/jcmm.15164

**Published:** 2020-06-12

**Authors:** Zunqiang Xiao, Linjun Hu, Liu Yang, Sheng Wang, Yuling Gao, Qiaojuan Zhu, Guo Yang, Dongsheng Huang, Qiuran Xu

**Affiliations:** ^1^ The Second Clinical Medical College of Zhejiang Chinese Medical University Hangzhou Zhejiang China; ^2^ The Medical College of Qingdao University Qingdao China; ^3^ The Key Laboratory of Tumor Molecular Diagnosis and Individualized Medicine of Zhejiang Province Zhejiang Provincial People’s Hospital (People’s Hospital of Hangzhou Medical College) Hangzhou China; ^4^ Department of Genetic laboratory Shaoxing Women and Children Hospital Shaoxing China; ^5^ Graduate Department Bengbu Medical College Bengbu China

**Keywords:** gastric cancer, lymphocytes, prognosis, TGFβ2, tumour infiltration

## Abstract

TGFβ2 is an essential regulator of immune cell functionality, but the mechanisms whereby it drives immune infiltration in gastric cancer remain uncertain. The Oncomine and Tumor Immunoassay Resource (TIMER) databases were used for assessing the expression of TGFβ2, after which TIMER was used to explore the relationship between TGFβ2 and tumour immune infiltration. Finally, we assessed how TGFβ2 expression correlated with the expression of a set of marker genes associated with immune infiltration using TIMER and GEPIA. We determined TGFβ2 expression to be significantly correlated with outcome in multiple types of cancer in the Cancer Genome Atlas (TCGA), with the effect being particularly pronounced in gastric cancer. Furthermore, elevated TGFβ2 expression was found to be significantly correlated with gastric cancer N staging, and with the expression of a variety of immune markers associated with particular immune cell subsets. These results indicate that TGFΒ2 is associated with patient outcome and tumour immune cell infiltration in multiple cancer types. This suggests that TGFβ2 is a key factor which governs immune cell recruitment to gastric cancer tumours, potentially playing a vital role in governing immune cell infiltration and thus representing a valuable prognostic biomarker in gastric cancer patients.

## 1 INTRODUCTION

Gastrics cancer (GC) remains among the deadliest forms of cancer, and it is particularly prevalent in East Asia.^1^ The poor prognosis of this cancer type is in part attributable to tumour metastasis.^2^ Immunological mechanisms regulate the development and progression of GC, and as such, many different immunotherapies have been proposed as a means of effectively treating this cancer type.^3^ In non‐small cell lung cancer, immunotherapies including inhibitors of cytotoxic T lymphocyte‐correlated antigen 4 (CTLA4), programmed death‐1 (PD‐1) and programmed death ligand‐1 (PD‐L1) have shown great promise.^4^ In GC, however, anti‐CTLA4 has shown poor efficacy in the clinic,^5^ and anti‐PD‐1 and anti‐PD‐L1 have shown only partial responses in advanced GC and colon cancer patients.^6^, ^7^, ^8^ The infiltration of immune cells into tumours is of particular relevance to patient outcome, with infiltration by tumour‐associated macrophages (TAMs) and neutrophils being of particular relevance to patient prognosis and tumour chemosensitivity.^9^ As such, there is a clear need to better clarify the immune phenotype of GC tumours and to better understand how immune cells regulate this type of cancer in order to better identify novel immunotherapy targets in GC.

Transforming growth factor beta (TGF‐β) is a cytokine particularly relevant to malignant tumour progression,^10^, ^11^, ^12^ with three family members—TGF‐β1, TGF‐β2 and TGF‐β3—playing non‐redundant roles in *vitro*.^13^ TGF‐β1 and TGF‐β2 have been shown to influence stromal and tumour cells in order to regulate tumour progression.^14^, ^15^ Most cancer cells lose the ability for TGF‐β to inhibit growth, thereby overcoming its suppressive activities while simultaneously enhancing its activities which favour tumour growth.^16^, ^17^ Indeed, TGF‐β1 has been shown to be independently predictive of both tumour stage and poor prognosis.^18^

TGF‐β signalling can induce profound immunosuppression, and it is secreted both by tumour cells and immune cells, in addition to other cells in the tumour microenvironment.^19^, ^20^ TGF‐β has the potential to drive the epithelial‐mesenchymal transition of tumour cells, thereby further enhancing tumour progression.^21^ When TGFβ signalling is inhibited, this has been found to prevent certain advanced tumours from metastasizing or progressing further,^22^, ^23^ while TGF‐β1 itself can impair immune cell responsiveness^24^, ^25^ while promoting angiogenesis.^26^

TGF‐β is a potent regulator of the tumour microenvironment, as it can regulate interactions between tumour, immune and stromal cells while simultaneously regulating cytokine production. Peripheral blood mononuclear cells (PBMCs) are key immune cells capable of secreting cytokines, and when they interact with cancer cells, this can either induce or impair a tumour‐specific immune response, thereby determining whether tumours undergo apoptotic death or are able to progress more rapidly.^20^, ^27^, ^28^ Tumour and PBMC interactions arise both through direct intercellular contact, and through cytokine‐dependent signalling pathways. Certain tumours have been found to induce the differentiation of naive peripheral CD4+ T cells into CD4+ CD25+ regulatory T cells via TGF‐β secretion,^29^, ^30^, ^31^ whereas other studies have found that the release of TNF‐α, interleukin (IL)‐1β, and IFN‐γ is elevated in certain cancer types, including in colon cancer upon interaction with lymphocytes.^32^ The mechanisms whereby TGFβ2 governs tumour progression and immune cell infiltration in GC, however, remain unclear.

Herein, we conducted a comprehensive assessment of the relationship between TGFβ2 and patient prognosis using databases including Oncomine, PrognoScan and Kaplan‐Meier plotter. We further investigated the link between TGFΒ2 and immune cell infiltration of tumours using the Tumor Immunoassay Resource (TIMER). Our results offer novel insights into the functional role of TGFβ2 in gastric cancer, thereby highlighting a potential mechanistic basis whereby TGFβ2 influences immune cell interaction with tumours.

## 2 MATERIALS AND METHODS

### 2.1 Oncomine database analysis

The Oncomine database compiled 86,733 samples and 715 gene expression data sets into a single comprehensive database designed to facilitate data mining efforts.^33^ We therefore used this database to assess the association between TGFβ2 expression and prognostic outcome in various tumour types (https://www.oncomine.org/resource/login.html).

### 2.2 PrognoScan database analysis

The PrognoScan database is designed to facilitate meta‐analyses of gene prognostic value by comparing the relationship between gene expression and relevant outcome including overall survival (OS) in a wide range of published cancer microarray data sets.^34^ We therefore used this database to assess the relationship between TGFβ2 expression and patient outcome (http://www.abren.net/PrognoScan/).

### 2.3 Kaplan‐Meier plotter analysis

The Kaplan‐Meier plotter offers a means of readily exploring the impact of a wide array of genes on patient survival in 21 different types of cancer, with large sample sizes for the breast (n = 6,234), ovarian (n = 2,190), lung (n = 3,452) and gastric (n = 1,440) cancer cohorts.^35^ We therefore used this database to explore the association between TGFβ2 expression and outcome in patients with gastric, breast, ovarian and lung cancer, analysing the impact of both clinicopathological factors and TGFβ2 on patient outcome in gastric cancer patients (http://kmplot.com/analysis/).

### 2.4 TIMER database analysis

TIMER (https://cistrome.shinyapps.io/timer/) is a database designed for analysing immune cell infiltrates in multiple cancers. This database employs pathological examination‐validated statistical methodology in order to estimate tumour immune infiltration by neutrophils, macrophages, dendritic cells, B cells and CD4/CD8 T cells.^36^ We initially employed this database to assess differences in TGFβ2 expression levels in particular tumour types using the TIMER database, and we then explored the association between this TGFβ2 expression and the degree of infiltration by particular immune cell subsets. We further conducted Kaplan‐Meier curve analyses to explore differences in patient survival as a function of gene expression or immune cell infiltration. Lastly, we assessed how TGFβ2 expression correlated with the expression of particular immune infiltrating cell subset markers.

### 2.5 GEPIA database analysis

GEPIA is an online database which facilitates the standardized analysis of RNA‐seq data from 9,736 tumour samples and 8,587 normal control samples in the TCGA and GTEx data sets (http://gepia.cancer‐pku.cn/index.html).^37^ We therefore employed this database to assess the link between TGFβ2 expression and patient prognosis in multiple tumour types, and we further assessed the link between TGFβ2 expression and the expression of particular markers associated with immune cell infiltration of tumours.

### 2.6 Statistical analysis

The PrognoScan, Kaplan‐Meier plotter, TIMER and GEPIA databases were used for generating survival plots in respective analyse, with data including either HR and *P‐*values or *P‐*values derived from a log‐rank test. Data from the Oncomine database are presented with information regarding ranking, fold‐change and *P‐*values. Spearman's correlation analyses were used to gauge the degree of correlation between particular variables, with the following *r* values being used to judge the strength of correlation: .00–.19 ‘very weak’, .20–.39 ‘weak’, .40–.59 ‘moderate’, .60–.79 ‘strong’, .80–1.0 ‘very strong’. *P* < .05 was the significance threshold.

## 3 RESULTS

### 3.1 Assessment of TGFβ2 expression in different cancer and normal tissues

**Figure 1:**
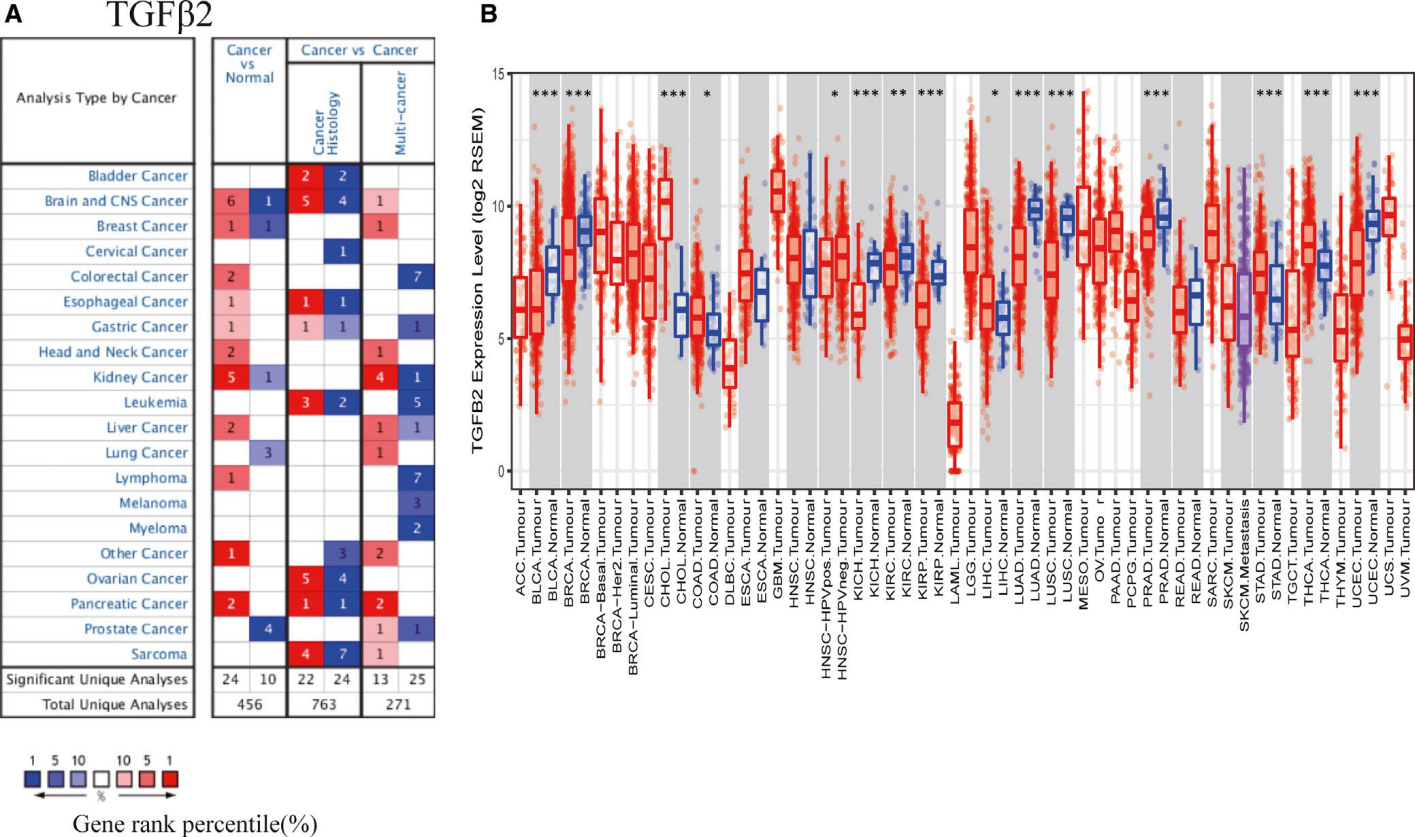
The expression level of TGFβ2 in different types of tumor tissues and normal tissues (A) The expression level of TGFβ2 in different types of tumor tissues and normal tissues in the Oncomine database. (*P* value is .001, fold change is 1.5, and gene ranking of all.) (B) The expression level of TGFβ2 in different types of tumor tissues and normal tissues in TIMER database (*P < .05, **P < .01, ***P < .001)

We first assessed the expression of TGFβ2 in multiple tumour and normal tissue types using the Oncomine database, revealing that expression of this gene was elevated relative to normal tissue controls for brain, breast, colorectal, oesophageal, rectal, gastric, head and neck, liver, renal and pancreatic cancers. We also found that relative to normal tissue controls, TGFβ2 expression was lower in brain, breast, renal, lung and prostate cancer tissues (Figure 1A). Detailed findings in particular tumour types are compiled in Table S1. We further used the TCGA and TIMER databases to assess how TGFβ2 expression differs in particular tumour types. We found that the expression of TGFβ2 was significantly elevated relative to normal controls in cholangiocarcinoma (CHOL), colon adenocarcinoma (COAD), liver hepatocellular carcinoma (LIHC), stomach adenocarcinoma (STAD) and thyroid carcinoma (THCA). In contrast, the expression of TGFβ2 was significantly below that in normal control tissues in bladder urothelial carcinoma (BLCA), breast invasive carcinoma (BRCA), kidney chromophobe (KICH), kidney renal papillary cell carcinoma (KIRP), kidney renal clear cell carcinoma(KIRC), lung adenocarcinoma (LUAD), lung squamous cell carcinoma (LUSC), prostate adenocarcinoma (PRAD) and uterine corpus endometrial carcinoma (UCEC). Differences between the expression of TGFβ2 in tumours and normal adjacent tissue samples in the TCGA data set are shown in Figure 1B.

### 3.2 The association between TGFβ2 expression and cancer patient prognosis

**Figure 2:**
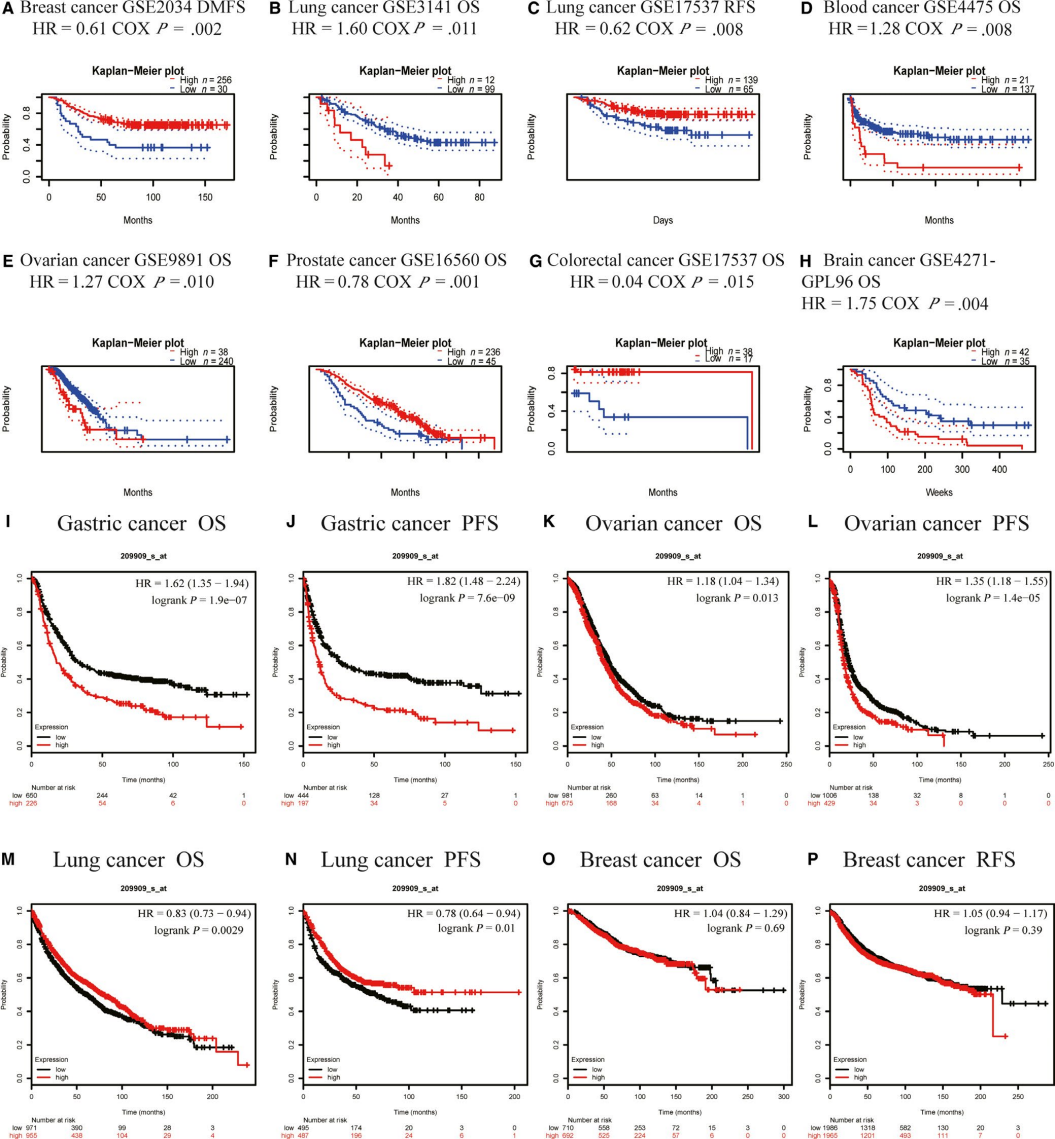
Correlation between TGFβ2 and prognosis of various types of cancer Correlation between TGFβ2 and prognosis of various types of cancer in the PrognoScan (A–H) Correlation between TGFβ2 and prognosis of various types of cancer in the Kaplan‐Meier plotter database (I–P). OS, overall survival; PFS, Kaplan‐Meier plotter database; RFS, recurrence‐free survival

We next explored the link between the expression of TGFβ2 and cancer patient outcome using the PrognoScan database (Tables S2–S5). We found that multiple cancer types exhibited a significant association between patient prognosis and TGFβ2 expression including breast, lung, blood, ovarian, prostate, brain and colon cancer (Figure 2A–H). We additionally employed the Kaplan‐Meier plotter database in order to assess how TGFβ2 expression relates to prognosis in a range of cancer types, revealing its elevation to be significantly linked with a poorer prognosis in gastric cancer (OS HR = 1.62, 95% CI = 1.35–1.98, *P* = 1.97e‐7; PFS HR = 1.82, 95% CI = 1.48–2.24, *P* = 7.6e‐9) and ovarian cancer (OS HR = 1.18, 95% CI = 1.04 to 1.34, *P* = .013; PFS HR = 1.35, 95% CI = 1.18–1.55, P = 1.4e‐5) (Figure 2I–L). However, we found reduced TGFβ2 expression to be correlated with poorer patient prognosis in lung cancer (OS HR = 0.83, 95% CI = 0.73–0.94, *P* = .0029; PFS HR = 0.78, 95% CI = 0.64–0.94, *P* = .01) (Figure 2M–N). There was not any significant relationship between the expression of TGFβ2 expression and the prognosis of breast cancer patients (Figure 2O–P). We further used the GEPIA database to assess how TGFΒ2 expression relates to patient prognosis, analysing 33 TCGA cancer types and revealing that TGFβ2 expression correlated both with OS and DFS in ACC, LGG, STAD (Figure [Link], [Link], [Link]). These results thus clearly demonstrate that TGFβ2 expression significantly correlated with poorer outcome in multiple tumour types.

### 3.3 Elevated TGFβ2 expression is linked to prognosis in gastric cancer patients exhibiting lymphatic metastasis

As we found TGFβ2 expression to be linked with poor gastric cancer patient prognosis, we next explored the underlying mechanisms via using the Kaplan‐Meier plotter database to assess the relationship between TGFβ2 expression and patient clinicopathological findings. We found that TGFβ2 expression correlated significantly with OS, DFS and with patient gender, stage, T stage, N stage, M stage, Lauren classification and differentiation, with the exception of stage 1 (Table 1). We further found TGFβ2 expression to correlate with each N stage, which corresponds to the degree of lymph node metastasis in gastric cancer patients. Such lymph node metastasis is the most common type of metastasis in gastric cancer patients and is directly linked with patient prognosis.^38^ With respect to the relationship between TGFβ2 and DFS in gastric cancer, N stage exhibited the highest HR (HR = 4.22 (1.56–11.44, *P* = .0020), suggesting that TGFβ2 expression has the potential to influence gastric cancer patient prognosis via influencing lymph node metastasis in these individuals.

**Table 1:** Kaplan‐Meier plotter to determine the effect of different clinicopathological factors on the expression of TGFβ2 gene and clinical prognosis in gastric cancer

Clinicopathological characteristics	Overall survival (n = 882)			Progression‐free survival (n = 646)		
	N	Hazard ratio	*P‐*value	N	Hazard ratio	*P‐*value
Sex						
Female	236	1.74 (1.20–2.54	.0033	201	2.11 (1.43–3.13)	.0001
Male	545	1.54 (1.23–1.94)	.0002	438	1.74 (1.37–2.22)	3.7e‐6
Stage						
1	67	1.60 (0.52–4.97)	.4108	60	1.72 (0.56–5.27)	.3398
2	140	2.29 (2.16–4.53)	.0146	131	2.01 (1.10–3.67)	.0199
3	305	0.81 (0.61–1.09)	.1638	186	1.93 (1.31–2.83)	.0007
4	148	2.31 (1.55–3.44)	2.2e‐5	141	2.23 (1.51–3.31)	5.0E‐5
Stage T						
2	241	2.63 (1.67–4.13)	1.4e‐5	239	2.70 (1.75–4.17)	3.1e‐6
3	204	1.52 (1.06–2.18)	.0229	204	1.61 (1.13–2.29)	.0078
4	38	5.08 (1.47–17.56)	.0047	39	3.24 (1.21–8.69)	.0138
Stage N						
0	74	3.98 (1.48–10.74)	.0032	72	4.22 (1.56–11.44)	.0020
1	225	1.99 (1.32–3.00)	.0008	222	2.04 (1.38–3.02)	.0003
2	121	1.69 (0.93–3.06)	.0835	125	1.66 (1.06–2.61)	.0254
3	76	2.93 (1.70–5.07)	6.0e‐5	76	3.00 (1.72–5.22)	5.0e‐5
1+2+3	422	2.04 (1.56–2.66)	1.2e‐7	423	2.15 (1.66–2.79)	3.1E‐9
Stage M						
0	444	2.18 (1.63–2.90)	4.9e‐8	443	2.15 (1.64–2.83)	1.6E‐8
1	56	1.67 (0.92–3.05)	.0902	56	1.91 (1.02–3.58)	.0391
Lauren classification						
Intestinal	320	1.52 (1.04–2.21)	.0287	263	1.91 (1.33–2.75)	.0004
Diffuse	241	2.28 (1.48–3.51)	.0001	241	2.28 (1.48–3.51)	.0001
Differentiation						
Poor	165	1.78 (1.12–2.83)	.0129	165	1.78 (1.12–2.83)	.0129
Moderate	67	1.96 (1.03–3.75)	.0377	67	1.96 (1.03–3.75)	.0377

### 3.4 TGFβ2 expression correlated with immune cell infiltration in gastric cancer

**Figure 3:**
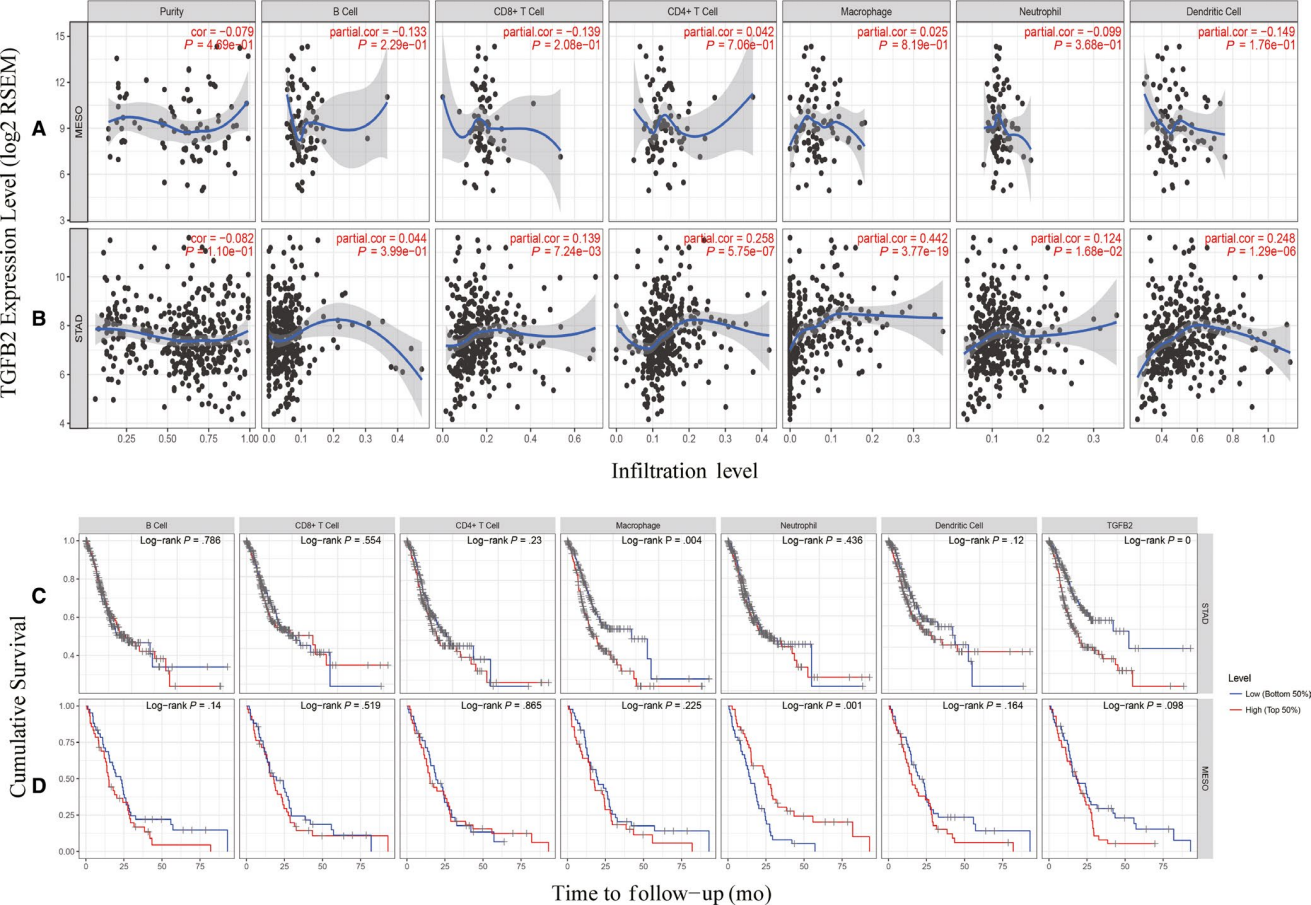
TGFβ2 expression is correlated with the level of immune infiltration in Stomach adenocarcinoma (STAD) and Mesothelioma (MESO). (A) TGFβ2 expression is correlated with the level of immune infiltration in Mesothelioma (MESO). (B) TGFβ2 expression is correlated with the level of immune infiltration in Stomach adenocarcinoma (STAD). (C) Kaplan‐Meier plots of immune infiltration and TGFβ2 expression levels in Stomach adenocarcinoma (STAD). (D) Kaplan‐Meier plots of immune infiltration and TGFβ2 expression levels in Mesothelioma (MESO)

In cancer patients, survival and lymph node metastasis are independently predicted by the frequency of lymphocytes infiltrating into the tumour.^39^, ^40^, ^41^ As such, we next explored the relationship between TGFβ2 expression and the degree of immune cell infiltration into 39 tumour types using the TIMER database (Figure S2). We found that there was a significant correlation between TGFβ2 expression and the tumour purity in 24 cancer types, and between TGFβ2 expression and B cell infiltration in 14 cancer types. There were additional correlations between TGFβ2 and the levels of CD8+T cell infiltration in 19 cancer types, CD4+T cell infiltration in 21 cancer types, macrophage infiltration in 23 cancer types, neutrophil infiltration in 23 cancer types, and dendritic cell infiltration in 23 cancer types. There was no significant association between TGFβ2 levels and B cell, CD4+T cell, CD8+T cell, macrophage, neutrophil or dendritic cell infiltration in mesothelioma (MESO) (Figure 3A). Similarly, there was no such relationship between levels of TGFβ2 and tumour purity in stomach adenocarcinoma (STAD), whereas in this same tumour type, the expression of TGFβ2 was significantly associated with levels of CD8+ T cells (R = .139, *P = *7.24e‐03), CD4+T cells (R = .258, *P *= 5.75e‐07), macrophages (R = .442, *P *= 3.77e‐19), neutrophils (R = .124, *P *= 1.68e‐02) and dendritic cells (R = .248, *P *= 1.29e‐05), although there was no relationship with B cell levels (Figure 3B). We further generated Kaplan‐Meier plots using the TIMER database in order to explore the relationship between immune cell infiltration and TGFβ2 expression in MESO and STAD. We found macrophage infiltration (*P *= .004) and TGFβ2 expression (*P *< .001) to significantly correlate with STAD prognosis (Figure 3C), whereas no significant correlation between prognosis and immune cell infiltration (*P *= .004) or TGFβ2 expression (*P *< .001) was observed in MESO (Figure 3D). This suggests that TGFβ2 plays a strong role in regulating immune cell infiltration in gastric cancer, with a particularly strong effect on macrophage infiltration.

### 3.5 Assessment of the correlation between TGFΒ2 and immune marker expression

**Figure 4:**
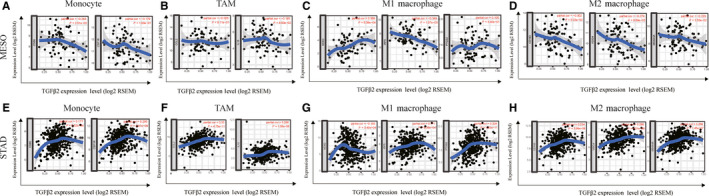
Correlation analysis between TGFΒ2 expression and immunological marker set in adenocarcinoma (STAD) and Mesothelioma (MESO). (A–D) Scatterplots of correlations between TGFB2 expression and gene markers of monocytes (A), TAMs (B), and M1 (C) and M2 macrophages (D) in MESO. (E–H) Scatterplots of correlations between TGFΒ2 expression and gene markers of monocytes (E), TAMs (F), and M1 (G) and M2 macrophages (H) in STAD

We next further explored the link between TGFβ2 expression and levels of immune cell infiltration based on sets of immunological markers in STAD using the TIMER and GEPIA databases, with MESO serving as a control group. Specifically, we assessed the correlation between TGFβ2 expression and levels of parkers for particular cell subsets including CD8+ T cells, total T cells, B cells, monocytes, TAMs, M1 and M2 macrophages, neutrophils, NK cells, DCs, Th1 cells, Th2 cells, Tfh cells, Th17 cells, Tregs and exhausted T cells. We adjusted these results based on tumour purity, revealing a significant correlation between TGFβ2 expression and monocyte markers (CD86, CD115), TAM markers (CCL2, IL10), M1 macrophage markers (INOS, IRF5, COX2), M2 macrophage markers (CD163, VSIG4, MS4A4A), neutrophils markers (CD11b, CD66b), NK cell markers (KIR2DL4), DC markers (BCDA‐A, BDCA‐4, CD11C), Th1 markers (STAT4), Th2 markers (GATA3, STAT5A), Tfh markers (BCL6), Th17 markers (STAT3) and Treg markers (CCR8, STAT5B, TGFβ1) in STAD (Table 2). In contrast, TGFβ2 expression correlated with just 10 of these markers in MESO (Table 2). TGFβ2 expression was correlated with that of the majority of monocyte, TAM, M1 and M2 macrophage markers in STAD (Table 2). In particular, it was significantly correlated with monocyte markers (CD86, CD115), TAM markers (CCL2, IL10), M1 macrophage markers (INOS, IRF5, COX2) and M2 macrophage markers (CD163, VSIG4, MS4A4A) in STAD (*P* < .0001; Figure 4A–H). We therefore further assessed the relationship between TGFβ2 expression and these markers in STAD using the GEPIA database revealing similar correlations between TGFβ2 and markers of monocytes, TAMs, and M1 and M2 macrophages to those in TIMER (Table 3). This suggests that in STAD, TGFβ2 may be capable of regulating the polarization of macrophages. Elevated TGFβ2 expression is also associated with increased DC infiltration in STAD, and consistent with this, the DC markers BDCA‐1, BDCA‐4 and CD11c were correlated with the expression of TGFβ2 expression. This indicates that TGFβ2 is closely linked with tumour DC penetration. DCs are able to increase levels of tumour metastasis via enhancing Treg responses and suppressing CD8+ T cell cytotoxicity.^42^ Further work will be necessary in order to establish whether TGFβ2 plays a key role in regulating DC infiltration and tumour metastasis. We further observed that there was a significant correlation between TGFβ2 and markers of Tregs and exhausted T cells including CCR8, STAT5B, TGFβ, TIM‐3 (Table 2), indicating that TGFβ2 may play a role in immune escape in gastric cancer, although further work will be needed to confirm the mechanisms underlying such escape.

**Table 2:** Correlation analysis between TGFβ2 and relate genes and markers of immune cells in TIMER

Description	Gene markers	STAD				MESO			
		None		Purity		None		Purity	
		Cor	*P*	Cor	*P*	Cor	P	Cor	*P*
CD8+T cell	CD8A	.117	.017	.116	.024	.135	.211	−.079	.469
	CD8B	.111	.023	.116	.024	.152	.160	−.163	.136
T cell(general)	CD3D	.053	.278	.041	.430	.146	.177	−.169	.136
	CD3E	.065	.187	.048	.352	.166	.124	−.197	.071
	CD2	.111	.023	.109	.034	.197	.067	−.226	.038
B cell	CD19	.129	*	.098	.057	.048	.661	−.073	.505
	CD79A	.142	*	.116	.024	.082	.450	−.100	.360
Monocyte	CD86	.176	**	.117	**	.227	.035	−.243	.025
	CD115(CSF1R)	.306	***	.295	***	.146	.176	−.179	.100
TAM	CCL2	.316	***	.330	***	.037	.736	−.026	.811
	CD68	.053	.279	.06	.246	.341	**	−.349	**
	IL10	.271	***	.288	***	.188	.080	−.185	.089
M1 Macrophage	INOS(NOS2)	−.180	**	−.183	**	.384	**	−.369	***
	IRF5	.170	**	.171	**	.362	**	−.349	*
	COX2(PTGS2)	.332	***	.329	***	.095	.382	.105	.340
M2 Macrophage	CD163	.237	***	.234	***	.261	.015	−.302	*
	VSIG4	.268	***	.296	***	.275	.010	−.279	*
	MS4A4A	.257	***	.266	***	.167	.121	−.229	.035
Neutrophils	CD66 b(CEACAM8)	.016	.743	.265	***	.047	.669	.062	.575
	CD11b(ITGAM)	.260	***	.204	***	.172	.112	−.163	.135
	CCR7	.216	***	.017	.744	.121	.264	−.146	.182
Natural killer cell	KIR2DL1	.087	.075	.074	.153	.230	.032	−.248	.021
	KIR2DL3	.072	.143	.049	.344	.448	***	−.452	***
	KIR2DL4	−.122	.012	−.141	*	.466	***	−.481	***
	KIR3DL1	.079	.108	.072	.164	.373	**	−.391	**
	KIR3DL2	.008	.871	.004	.939	.212	.049	−.240	.027
	KIR3DL3	−.105	.032	−.118	.021	.243	.023	−.250	.211
	KIR2DS4	.018	.715	.001	.989	.204	.058	−.195	.073
Dendritic cell	HLA‐DPB1	.051	.299	.037	.471	.035	.746	.050	.649
	HLA‐DQB1	−.089	.070	−.114	.026	.125	.249	−.129	.239
	HLA‐DRA	−.038	.443	−.048	.353	.076	.486	−.097	.376
	HLA‐DPA1	.010	.837	.001	.992	.048	.656	−.072	.513
	BCDA‐1(CD1C)	.287	***	.292	***	.166	.125	.131	.234
	BDCA‐4(NRP1)	.502	***	.500	***	.321	*	.289	*
	CD11c(ITGAX)	.192	***	.182	**	.243	.024	−.243	.024
Th1	T‐bet(TBX21)	.081	.098	.075	.147	.298	.005	−.354	*
	STAT4	.219	***	.195	**	.143	.187	−.229	.035
	STAT1	−.032	.516	−.045	.378	.225	.036	−.236	.030
	IFN‐γ(IFNG)	−.081	.101	−.087	.092	.083	.447	−.093	.399
	TNF‐α(TNF)	.093	.059	.088	.086	.078	.473	.089	.416
Th2	GATA3	.253	***	.259	***	.054	.616	.074	.050
	STAT6	−.052	.294	−.051	.318	.204	.058	−.217	.046
	STAT5A	.172	**	.171	**	.147	.174	−.128	.241
	IL13	.078	.114	.074	.148	.178	.099	.170	.101
Tfh	BCL6	.387	***	.367	***	.052	.634	−.012	.915
	IL21	.016	.752	.012	.810	.132	.223	.129	.241
Th17	STAT3	.289	***	.266	***	.105	.332	−.105	.340
	IL17A	−.084	.088	−.092	.074	.056	.604	.077	.486
Treg	FOXP3	.052	.294	.033	.520	.093	.390	.064	.558
	CCR8	.209	***	.207	***	.020	.851	−.016	.884
	STAT5B	.370	***	.367	***	.103	.342	.072	.511
	TGFβ(TGFB1)	.381	***	.358	***	.052	.630	.057	.602
T cell exhaustion	PD‐1(PDCD1)	.065	.182	.065	.206	.043	.689	.058	.600
	CTLA4	.096	.050	.098	.057	.033	.764	.025	.828
	LAG3	.009	.858	−.004	.942	.197	.068	−.202	.063
	TIM‐3(HAVCR2)	.127	*	.131	.010	.252	.019	−.260	.016
	GZMB	−.084	.088	−.104	.042	.311	*	.333	.187

Cor, R value of Spearman’s correlation; None, correlation without adjustment. Purity, correlation adjusted by purity. *P < .01; **P < .001; ***P < .0001.

Abbreviations: MESO, mesothelioma; STAD, stomach adenocarcinoma; TAM, tumour‐correlated macrophage; Tfh, follicular helper T cell; Th, T helper cell; Treg, regulatory T cell.

**Table 3:** Correlation analysis between TGFβ2 and relate genes and markers of monocyte, TAM and macrophages in GEPIA

Description	Gene markers	STAD			
		Tumour		Normal	
		R	*P*	R	*P*
Monocyte	CD86	.3	***	−.28	.099
	CD115(CSF1R)	.42	***	.11	.52
TAM	CCL2	.37	***	.51	*
	CD68	.21	***	−.47	*
	IL10	−.4	***	−.06	.73
M1Macrophage	INOS(NOS2)	−.088	.076	.066	.7
	IRF5	.29	***	−.31	.067
	COX2(PTGS2)	.39	***	.76	***
M2Macrophage	CD163	.33	***	.64	***
	VSIG4	.37	***	.38	.024
	MS4A4A	.37	***	.4	.017

Cor, R value of Spearman’s correlation; None, correlation without adjustment. Purity, correlation adjusted by purity. *P < .01; **P < .001; ***P < .0001.

Abbreviations:MESO, mesothelioma; STAD, stomach adenocarcinoma; TAM, tumour‐correlated macrophage; Tfh, Follicular helper T cell; Th, T helper cell; Treg, regulatory T cell.

## 4 DISCUSSION

TGFβ2 is a transforming growth factor beta (TGFB) family cytokine, with members of this cytokine family playing broad regulatory roles and controlling key physiological processes including cell migration, proliferation and differentiation via signalling through type I and type II receptors (TGFβR1 and TGFβR2), with signals propagating via the downstream regulatory SMAD proteins. This TGFβ/SMAD pathway is frequently dysregulated in human cancer. TGFβ cytokines are capable of suppressing T cell growth in response to IL‐2. In this study, we found that TGFβ2 expression correlated with patient prognosis in several types of cancer, with a particularly strong correlation between high TGFβ2 expression and a poor STAD prognosis. This elevated TGFβ2 expression was also a reliable predictor of the presence of lymph node metastasis in GC patients, indicating that TGFβ2 may be a valuable prognostic indicator of metastatic progression in GC tumour types. We further found that the degree of TGFβ2 expression correlated with the expression of several different markers of immune cell subsets within tumours, thus highlighting a possible role for TGFβ2 in the immunological interactions in GC, making it a valuable biomarker worthy of further research in this type of cancer.

In this report, we assessed the expression of TGFβ2 as it related to the prognosis of 33 different types of cancers using the independent Oncomie and GEPIA databases, revealing clear differences between tumour and normal tissue expression of TGFβ2 in many cancers. Oncomine data revealed elevated TGFβ2 levels in brain, breast, colorectal, oesophageal, gastric, head and neck, renal, liver, pancreatic and lymphoma cancers relative to normal tissue, whereas in certain data sets TGFβ2 levels were lower in brain, breast, kidney, lung and prostate cancer (Figure 1A). TCGA data set analysis indicated that there was elevated TGFβ2 expression in CHOL, COAD, LIHC, STAD and thyroid THCA, whereas expression was decreased in BLCA, BRCA, KICH, KIRP, KIRC, LUAD, LUSC, PRAD and UCEC relative to adjacent controls (Figure 1B). Altered TGFβ2 expression in a range of different cancers may be due to the different means of data collection in different studies, or it may relate to differences in the underlying biological mechanisms. Across these databases, we consistently observed a correlation between elevated TGFβ2 expression and a poor GC prognosis. In the TCGA database, elevated TGFβ2 levels were correlated with a poorer outcome for patients with ACC, LGG and STAD. Similarly, the Kaplan‐Meier plotter database found elevated TGFβ2 to correlate with poor GC and ovarian cancer outcome (Figure 2I–L). Furthermore, elevated TGFβ2 correlated with poorer patient prognosis, as well as gender, stage, T stage, N stage, M stage, Lauren classification and differentiation. Elevated TGFβ2 expression in GC correlated with a higher N stage HR in PFS (Table 1). These results together thus suggest that TGFβ2 may have value as a GC prognostic biomarker.

An additional key finding in this study is that the expression of TGFβ2 correlated with the degree of immune infiltration in multiple cancer types, and particularly in GC. We found that TGFβ2 expression was moderately positively correlated with the degree of macrophage infiltration, and weakly positively correlated with the degree of CD8+, CD4+, DC and neutrophil infiltration in STAD (Figure 3A). We further found macrophage infiltration to be significantly associated with GC prognosis (Figure 3C) In addition, the correlation observed between TGFβ2 and the expression of certain immunological marker genes strongly suggests that in STAD tumours TGFβ2 can control immune cell infiltration and interactions within the tumour microenvironment. We observed a weak correlation between TGFβ2 and M1/M2 macrophage markers including PTGS2, IRF5, CD163, VSIG4 and MS4A4A (Table 3). This suggests that TGFβ2 play a role in regulating TAM polarization. We further found TGFβ2 levels in STAD to correlate with markers of Treg cells and T cell exhaustion (CCR8, STAT5B and TGFB1) (Table 2). This suggests that TGFβ2 can promote Treg responses to suppress T cell‐mediated immunity. Furthermore, we found that expression of TGFβ2 correlated with that of multiple T cell markers (Th1, Th2, Tfh and Th17) in STAD. This may correspond to the ability of TGFβ2 to regulate T cell responses in STAD. Together, these results highlight the ability of TGFβ2 to potentially regulate immune cell recruitment and activation in STAD.

In summary, TGFβ2 may be an important regulator of immune cell infiltration and a valuable prognostic biomarker in gastric cancer patients.

## CONFLICT OF INTEREST

The authors declared that they have no competing interests.

## AUTHORS' CONTRIBUTIONS

LH and ZX conceived the project and wrote the manuscript. SW, LY, QZ, YG and YG participated in data analysis. QX participated in discussion and language editing. DH reviewed the manuscript.

## Supporting information

Fig S1AW‐BN

Fig S1A‐X

Fig S1Y‐AV

Fig S2Y‐AF

Fig S2AG‐AK

Fig S2A‐H

Fig S2I‐P

Fig S2Q‐X

Tables S1‐S5

## Data Availability

The data that support the findings of this study are available in the cancer genome atlas(TCGA)at [https://portal.gdc.cancer.gov] and gene expression omnibus (GEO) at [https://www.ncbi.nlm.nih.gov/gds/], these databases are public databases.
