# Supplementary figures and images for: TGFβ2 is a prognostic‐related biomarker and correlated with immune infiltrates in gastric cancer
Source: J Cell Mol Med. 2020 Jun 12;24(13):7151–62. doi: 10.1111/jcmm.15164 (PMC7339175; doi:10.1111/jcmm.15164)

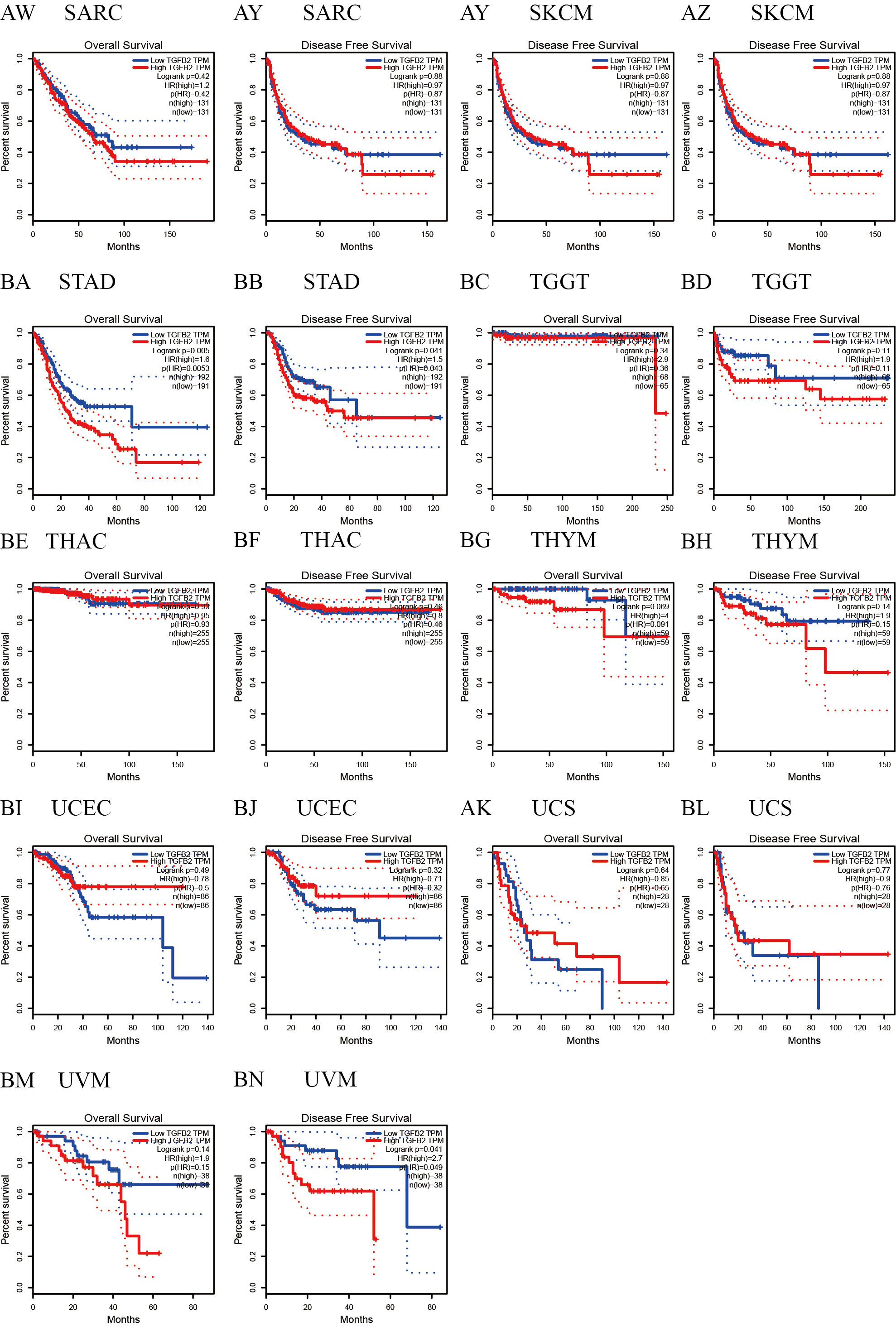

Supplement: Supplementary file 1 — Fig S1AW‐BN [file JCMM-24-7151-s001.tif]

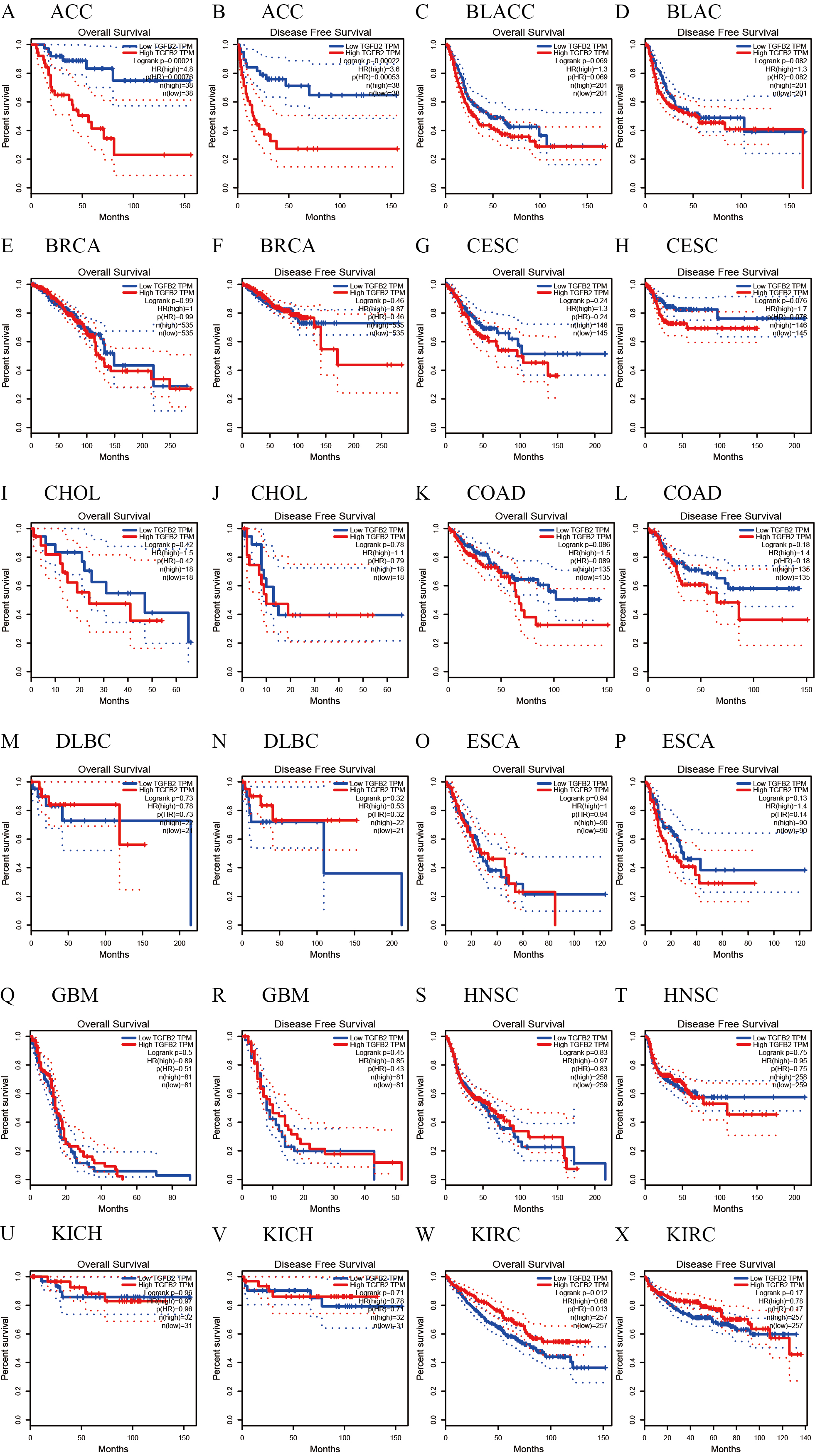

Supplement: Supplementary file 2 — Fig S1A‐X [file JCMM-24-7151-s002.tif]

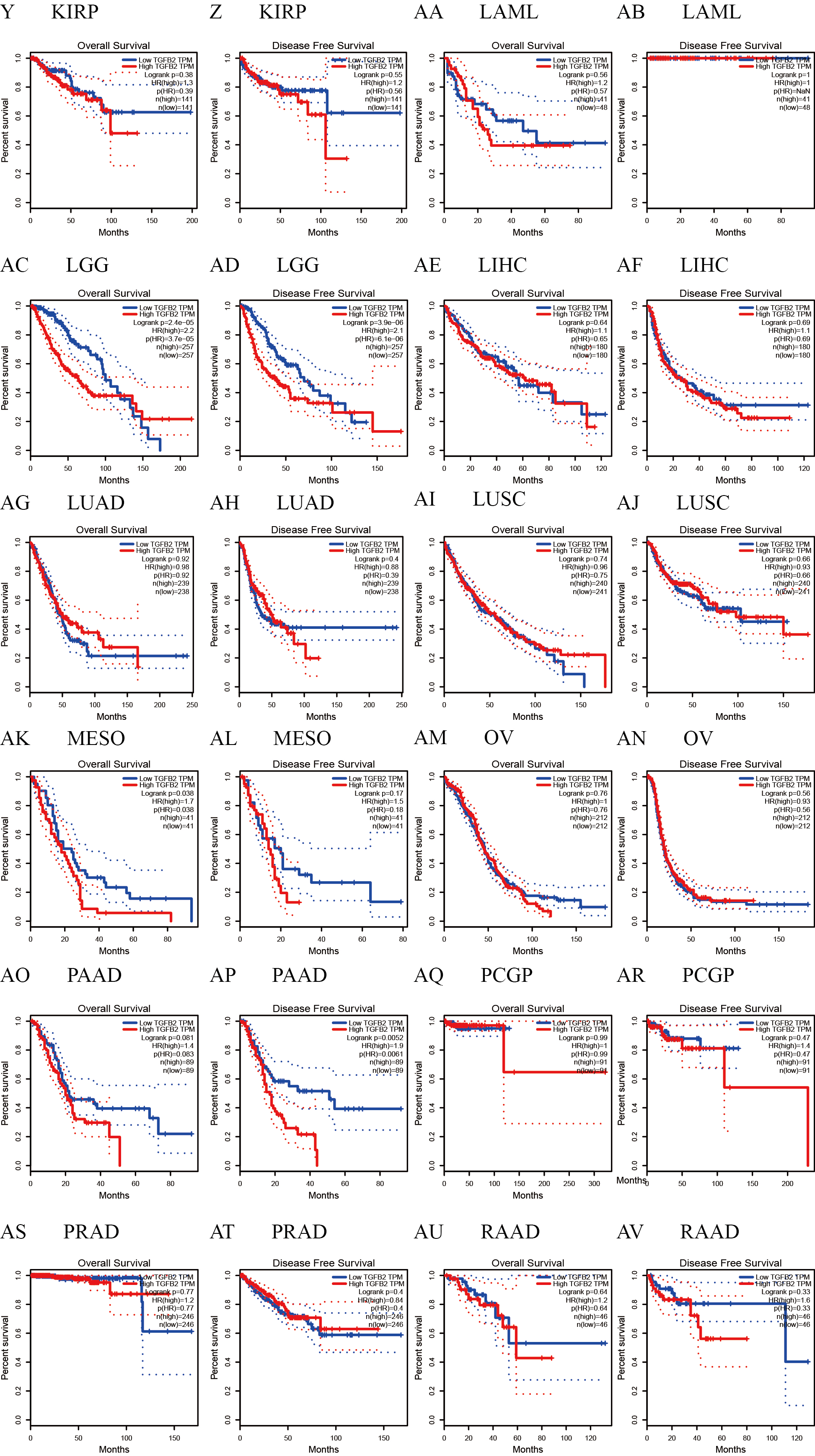

Supplement: Supplementary file 3 — Fig S1Y‐AV [file JCMM-24-7151-s003.tif]

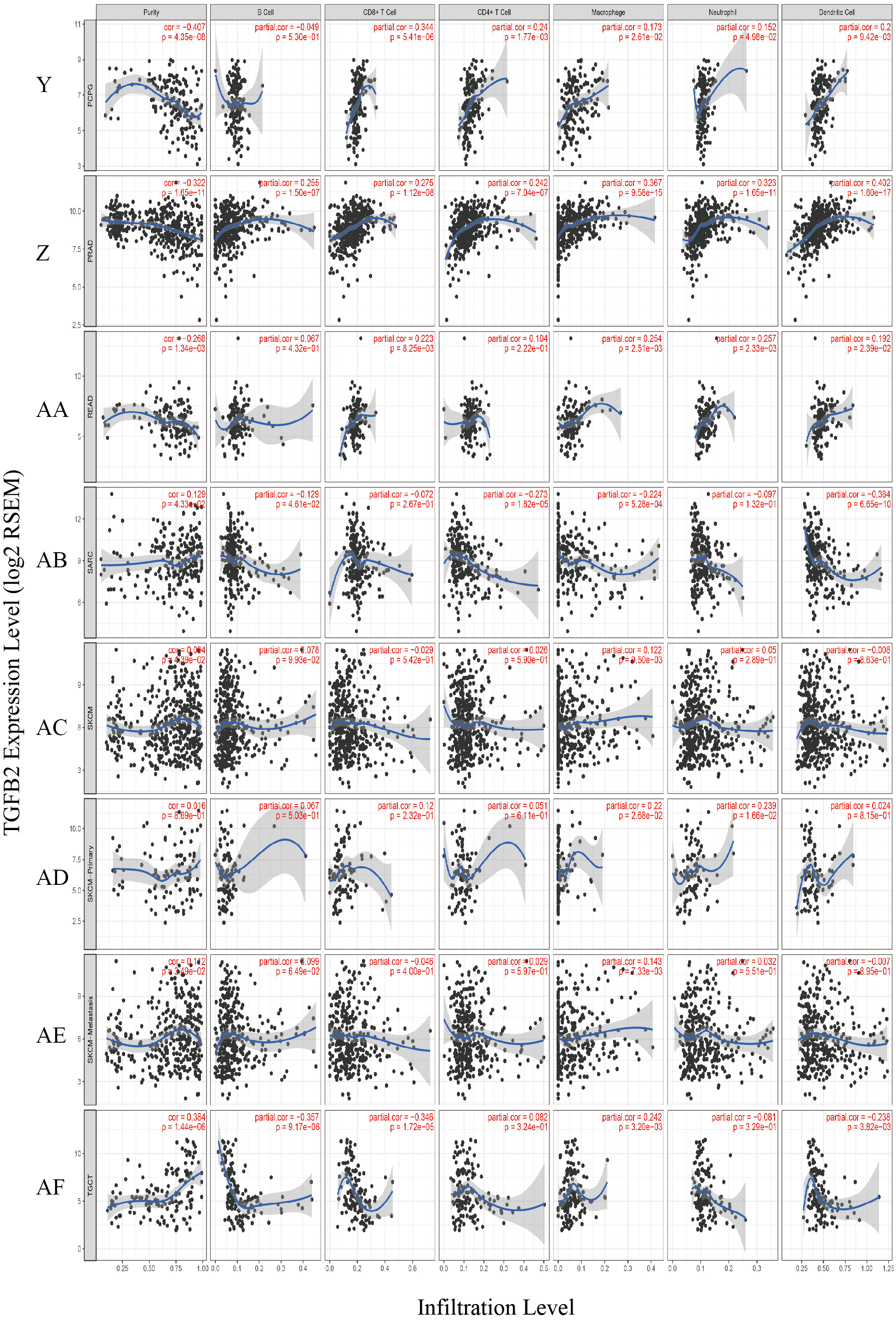

Supplement: Supplementary file 4 — Fig S2Y‐AF [file JCMM-24-7151-s004.tif]

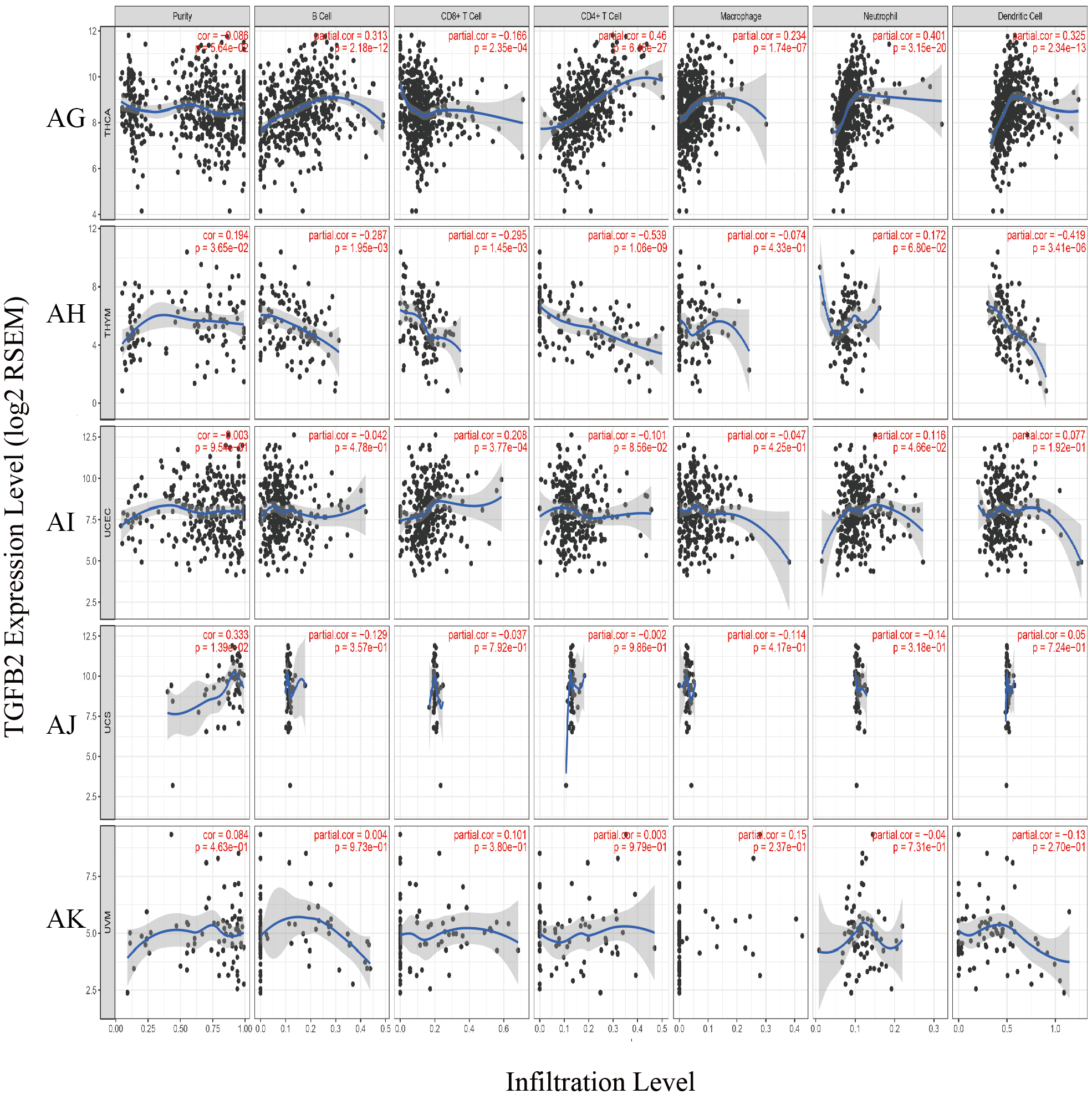

Supplement: Supplementary file 5 — Fig S2AG‐AK [file JCMM-24-7151-s005.tif]

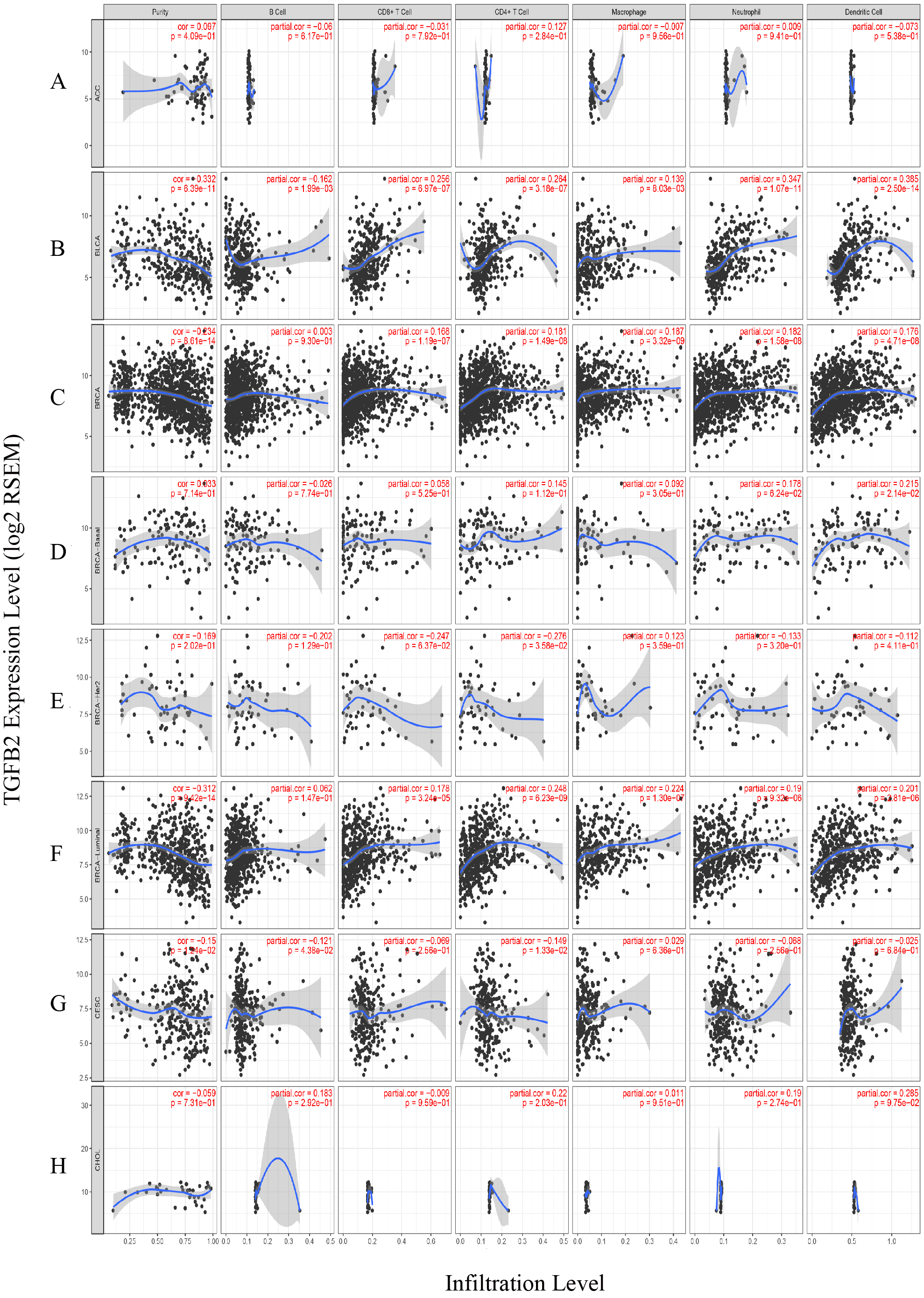

Supplement: Supplementary file 6 — Fig S2A‐H [file JCMM-24-7151-s006.tif]

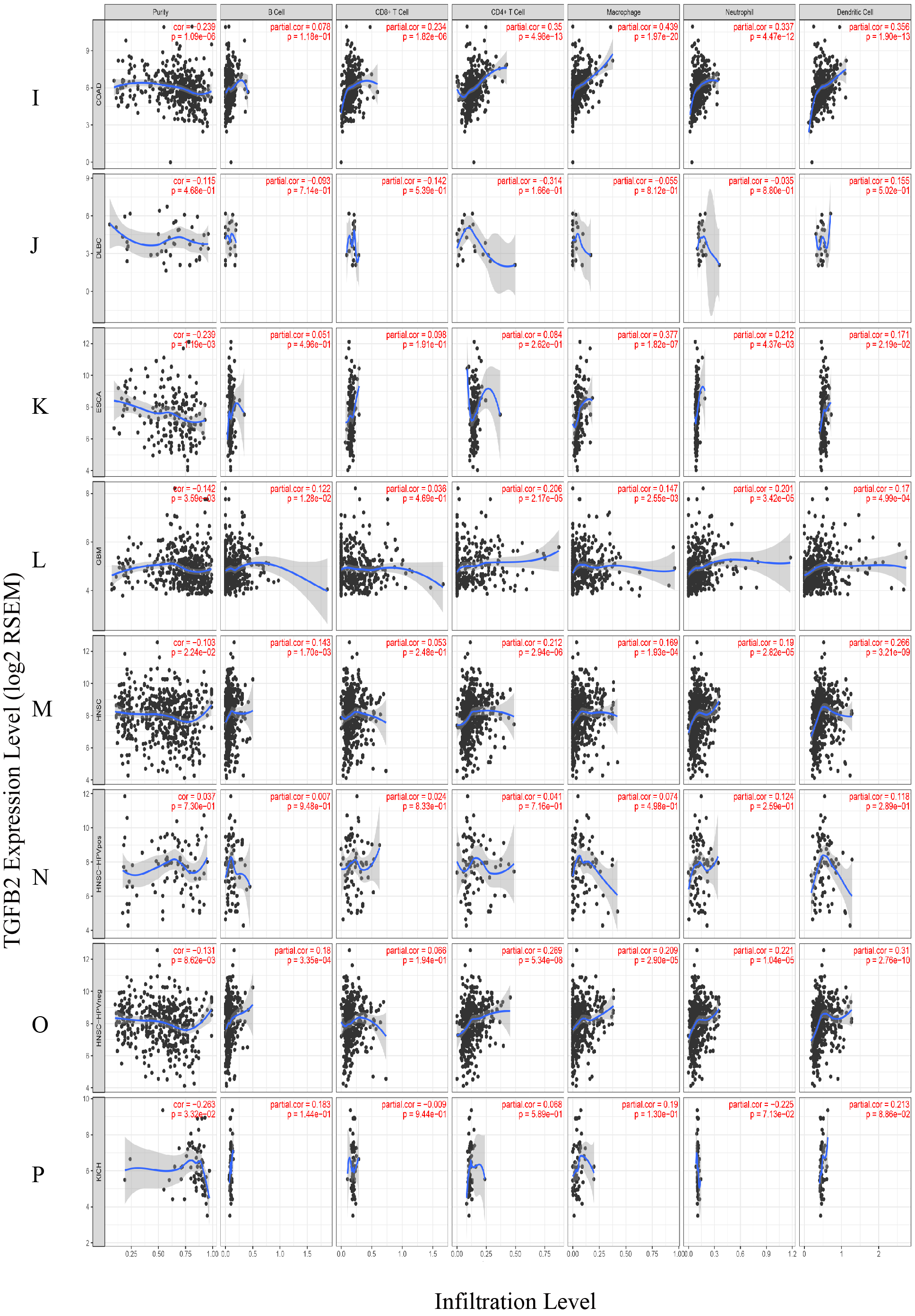

Supplement: Supplementary file 7 — Fig S2I‐P [file JCMM-24-7151-s007.tif]

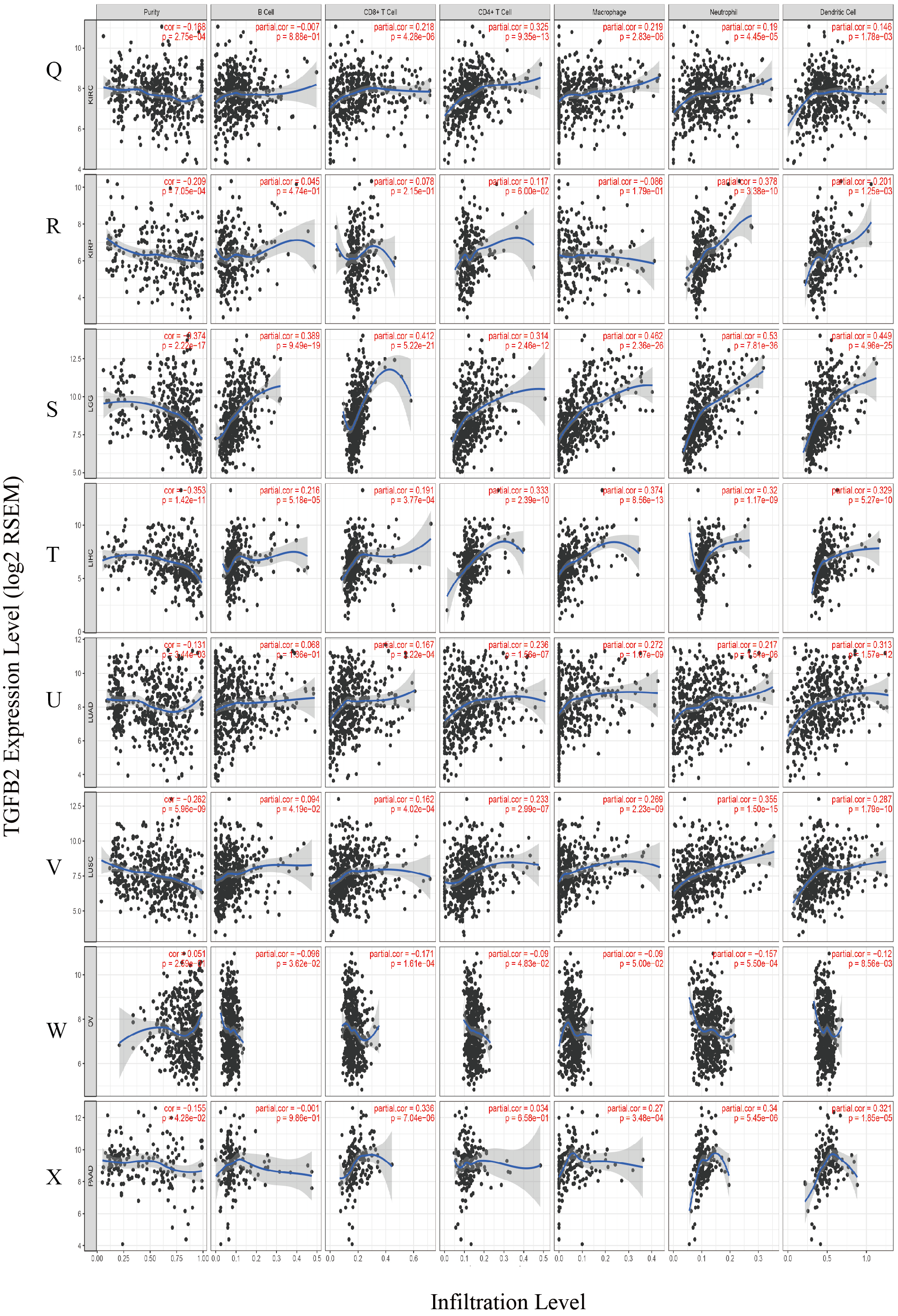

Supplement: Supplementary file 8 — Fig S2Q‐X [file JCMM-24-7151-s008.tif]
