# Supplementary material for: TGFβ2 is a prognostic‐related biomarker and correlated with immune infiltrates in gastric cancer
Source: J Cell Mol Med. 2020 Jun 12;24(13):7151–62. doi: 10.1111/jcmm.15164 (PMC7339175; doi:10.1111/jcmm.15164)

Supplementary Table 1. TGFβ2 expression in cancers vs normal tissue in oncomine database

| Cancer | Cancer type | *P*-value | Fold change | Rank (%) | Sample | Reference (PMID) |
| --- | --- | --- | --- | --- | --- | --- |
| Brain and CNS | Classic Medulloblastoma | 3.03E-11 | 18.906 | 2% | 60 | 11807556 |
|  | Desmoplastic Medulloblastoma | 2.51E-4 | 20.691 | 3% | 18 | 11807556 |
|  | Glioblastoma | 8.65E-5 | 3.103 | 3% | 32 | 15827123 |
|  | Glioblastoma | 8.45E-17 | 3.363 | 2% | 104 | 16616334 |
|  | Anaplastic Astrocytoma | 1.18E-4 | 1.879 | 10% | 42 | 16616334 |
|  | Glioblastoma | 2.50E-7 | 1.539 | 4% | 84 | 18565887 |
| Breast | Ductal Breast Carcinoma in Situ | 8.15E-5 | 2.114 | 2% | 25 | 19187537 |
| Colorectal | Colon Carcinoma | 6.45E-10 | 2.659 | 2% | 15 | 20957034 |
|  | Rectal Adenocarcinoma | 2.20E-17 | 2.158 | 8% | 130 | 20725992 |
| Esophageal | Esophageal Adenocarcinoma | 4.84E-4 | 10.341 | 8% | 13 | 16952561 |
| Gastric | Gastric Mixed Adenocarcinoma | 2.21E-4 | 1.760 | 6% | 35 | 19081245 |
| Head and Neck | Tongue Squamous Cell Carcinoma | 2.23E-4 | 1.655 | 4% | 38 | 18254958 |
|  | Oral Cavity Squamous Cell Carcinoma | 4.71E-7 | 1.668 | 9% | 79 | 21853135 |
| Kidney | Renal Pelvis Urothelial Carcinoma | 1.24E-14 | 4.313 | 1% | 31 | 16115910 |
|  | Chromophobe Renal Cell Carcinoma | 1.63E-14 | 4.601 | 1% | 29 | 16115910 |
|  | Papillary Renal Cell Carcinoma | 1.92E-14 | 4.038 | 2% | 34 | 16115910 |
|  | Renal Oncocytoma | 3.24E-16 | 4.183 | 2% | 35 | 16115910 |
|  | Clear Cell Renal Cell Carcinoma | 8.78E-16 | 3.914 | 3% | 46 | 16115910 |
| Liver | Hepatocellular Carcinoma | 1.61E-5 | 1.911 | 5% | 45 | 17393520 |
| Lymphoma | Diffuse Large B-Cell Lymphoma | 9.16E-4 | 3.171 | 3% | 32 | 10676951 |
| Others | Uterine Corpus Leiomyoma | 9.02E-9 | 2.369 | 1% | 77 | 19622772 |
| Pancreatic | Pancreatic Ductal Adenocarcinoma | 1.76E-5 | 1.961 | 1% | 14 | 16103885 |
|  | Pancreatic Ductal Adenocarcinoma | 9.09E-11 | 2.714 | 3% | 78 | 19260470 |

Supplementary Table 2. Relation between TGFβ2 expression and patient prognosis of different cancer(Disease Specific Survival) in Prognoscan database.


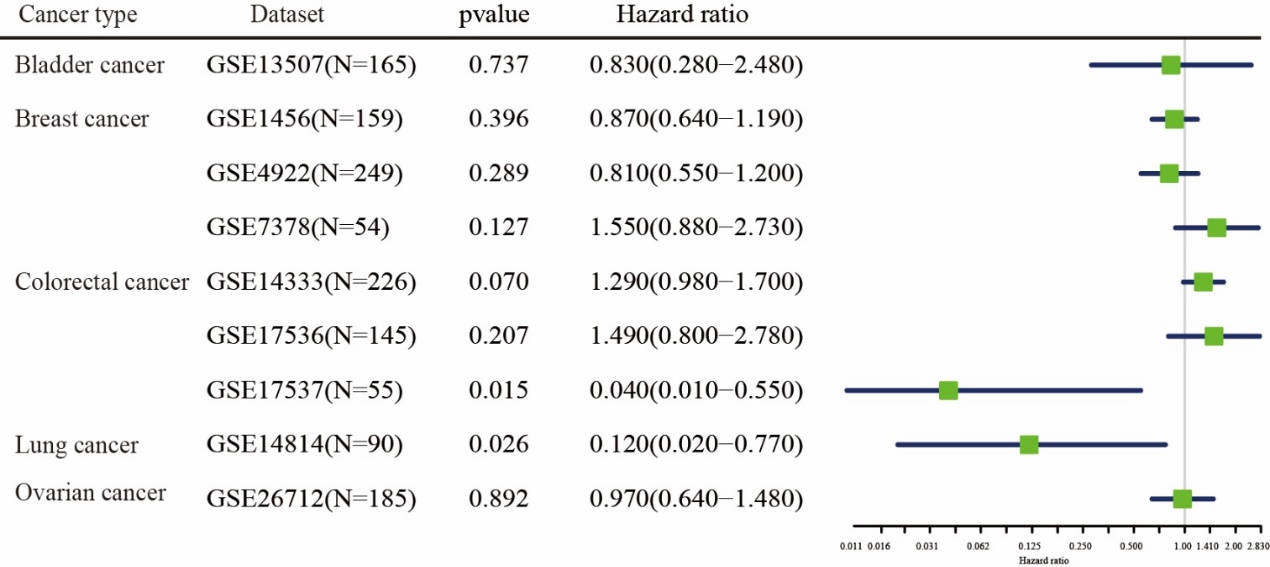


Supplementary Table 3. Relation between TGFβ2 expression and patient prognosis of different cancer(Distant Metastasis Free Survival) in Prognoscan database.


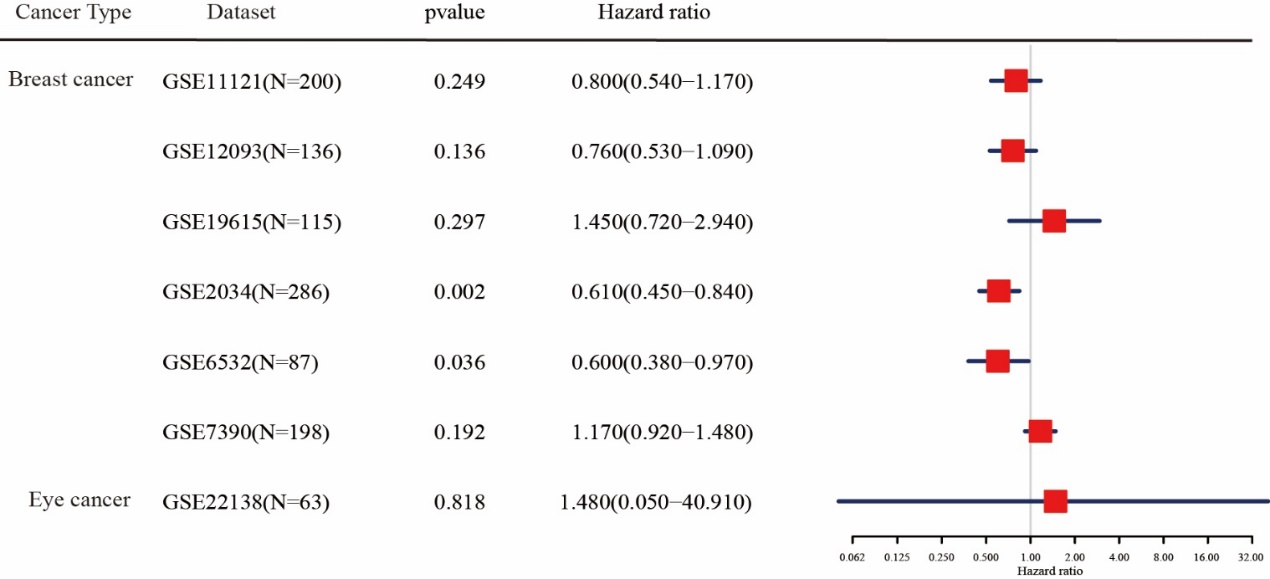


Supplementary Table 4. Relation between TGFβ2 expression and patient prognosis of different cancer(Overall Survival) in Prognoscan database.


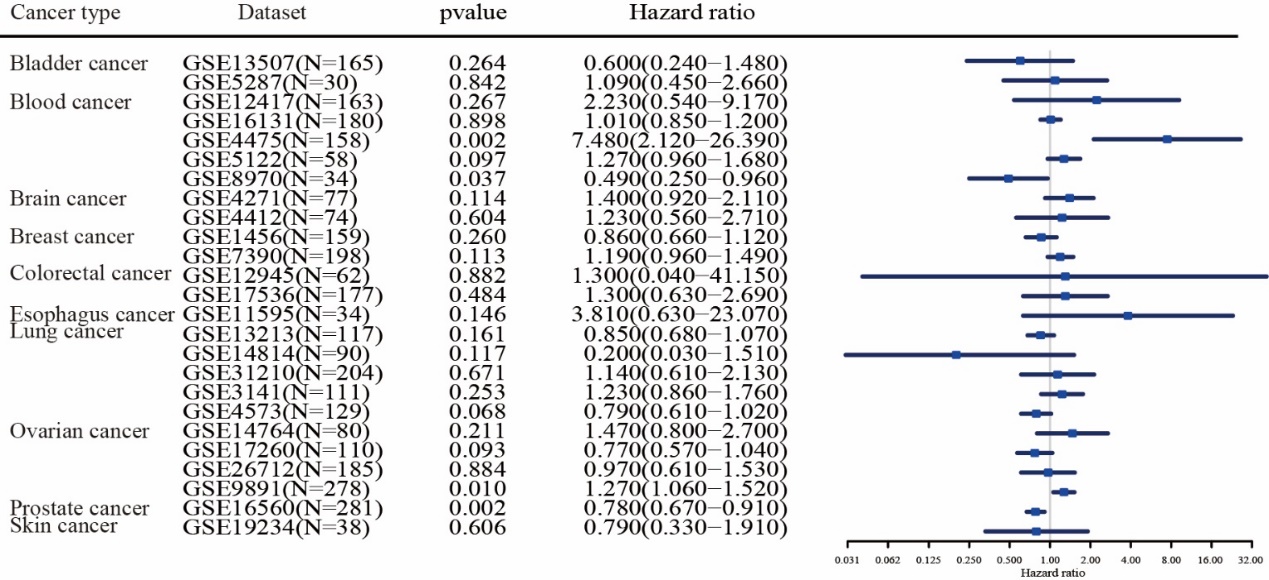


Supplementary Table 5. Relation between TGFβ2 expression and patient prognosis of different cancer(Relapse Free Survival) in Prognoscan database.


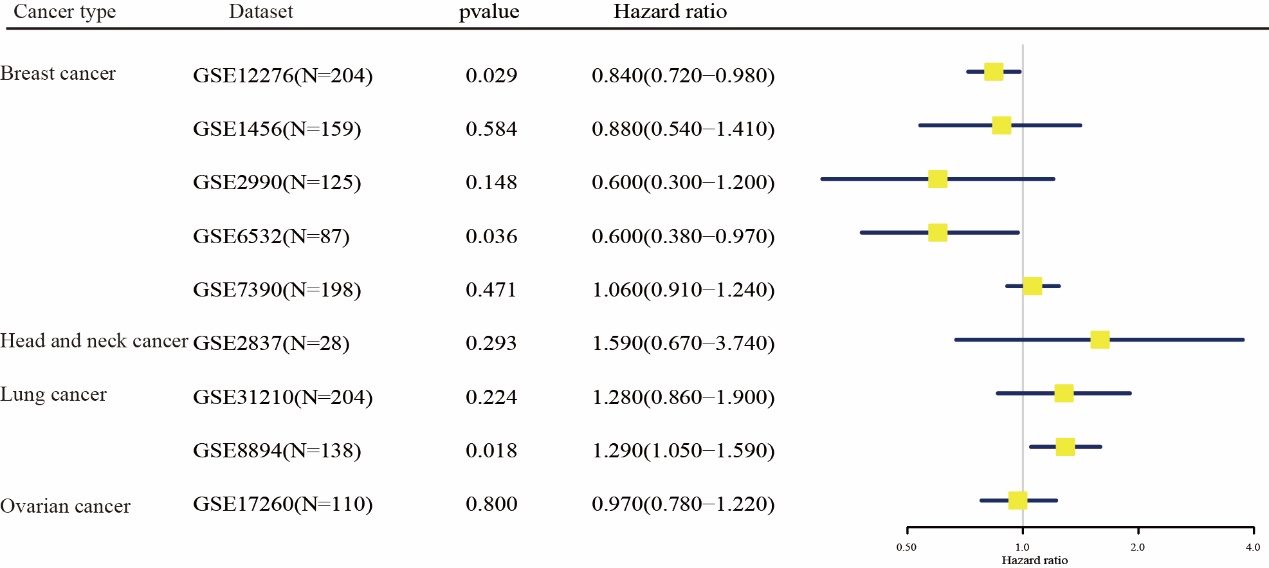

Supplement: Supplementary file 9 — Tables S1‐S5 [file JCMM-24-7151-s009.docx]
